# A Joint Space-Time Array for Communication Signals-Based on a Moving Platform and Performance Analysis

**DOI:** 10.3390/s18103388

**Published:** 2018-10-10

**Authors:** Bin Yang, Cheng Wang, Bin Yang, Jiexin Yin

**Affiliations:** 1 Zhengzhou Information Science and Technology Institute, Zhengzhou 450002, China; forestsyoung@outlook.com (B.Y.); Cindyin0807@163.com (J.Y.); 2 National Digital Switching System Engineering and Technology Research Center, Zhengzhou 450002, China; ybmailbox@126.com

**Keywords:** moving array, joint space-time processing, Doppler shift, communication signal

## Abstract

A joint space-time array for communication signals is constructed in this paper to settle the contradiction between the performance of angle estimation and the array aperture. It introduces Doppler information caused by platform motion into the signal processing to obtain favorable performance with limited array aperture. We analyze the theoretical performance in the aspects of distinguishable source number, spatial resolution and Cramér-Rao bound (CRB), respectively. Both theoretical analysis and simulation results demonstrate that the proposed space-time array can give rise to a significant enhancement in achievable array performance.

## 1. Introduction

Direction of arrival (DOA) estimation is an important branch of array signal processing. The performance of DOA estimators mainly depends on the aperture of the array. In the modern electromagnetic environment, antenna arrays may be deployed on moving platforms (e.g., airplanes, satellites, ships) to enhance their maneuverability [1]. However, apertures of those arrays are generally restricted by the size of platform they attached to, which could lead to degradation on DOA estimation performance if not of a suitable size [2].

Various methods have been proposed to solve this problem, for instance, high order cumulant [3], cyclostationarity [4] and non-circularity [5] of the signal were exploited to improve DOA performance. These techniques rely on temporal properties of the signal waveform, so they are only suitable for specific signals. Algorithms based on intelligent processing were also developed for DOA estimation. In [6], a deep neural network (DNN) was designed and satisfactory DOA performance were observed. The above algorithms have their respective advantages, but they are not effective when array apertures are limited.

In other studies, some compressive sensing (CS)-based DOA estimation algorithms take the moving array condition into consideration. Reference [7] formulated the DOA estimation problem as a Multiple Measurement Vectors (MMV) problem and solved it by minimizing a mixed Euclidean norm approximation. Liu [8] also adopted Euclidean norm minimization approach to estimate the DOA, but divided the range of interest into low-resolution grids, which conspicuously reduced the computation complexity. Reference [9] employed a second order core (SOC) programming method to solve the optimization problem. However, CS technique algorithms are mainly designed to deal with sample limitation problems. They cannot figure out the raised problem where a limited array aperture is available.

Alternatively, the synthetic aperture (SA) technique, which utilizes Doppler information to improve DOA performance, has received increasing attention in moving array systems. This technique was firstly used in cooperative scenarios for radar imaging [10,11] and then extended to non-cooperative acoustic processing [12,13,14]. However, the applications of the SA technique in communication signal processing have not been addressed often. Unlike radar or sonar where the signal source is under the control of the receiver, communication signals have complicated modulation patterns and unknown data sequences, which generate unpredictable phase fluctuations in the signal Doppler information. Researchers then modified the SA technique and proposed space-time signal processing methods for the purpose of applying the SA technique to communication signals [15]. These space-time processing methods mainly focus on the scenario where a static array detects a moving target [16]. Reference [17] analyzed the performance of such space-time signal models.

To the best of our knowledge, methods intended for communication signals with moving array have not received much attention. On the basis of the existing SA techniques, we propose a space-time array model for moving arrays in this paper. Its performance is thoroughly analyzed for comparison with a conventional array. First, the distinguished source number is derived under different conditions. Then we utilize the Euclidean norm to analyze the array spatial resolution enhancement. Finally, the Cramér-Rao bound (CRB) is computed in order to evaluate the estimation accuracy. The theoretical results are further discussed so as to cope with the parameter selection problem for space-time arrays. In contrast to conventional arrays, the proposed space-time array introduces Doppler information into the DOA estimation and manifests superior performance. Numeric simulations are also provided to demonstrate its effectiveness.

The paper is organized as follows: Section 2 presents some assumptions and the space-time array model. In Section 3, we evaluate the performances of space-time array through distinguishable source number, spatial resolution and CRB of such three aspects. The performance comparison and some theoretical conclusions are also involved in this section. In Section 4, several numerical simulations are presented to verify the theoretical derivation. Finally, Section 5 presents our conclusions and identifies future work.

## 2. Scenario Description and Space-Time Array Modeling

**Figure 1:**
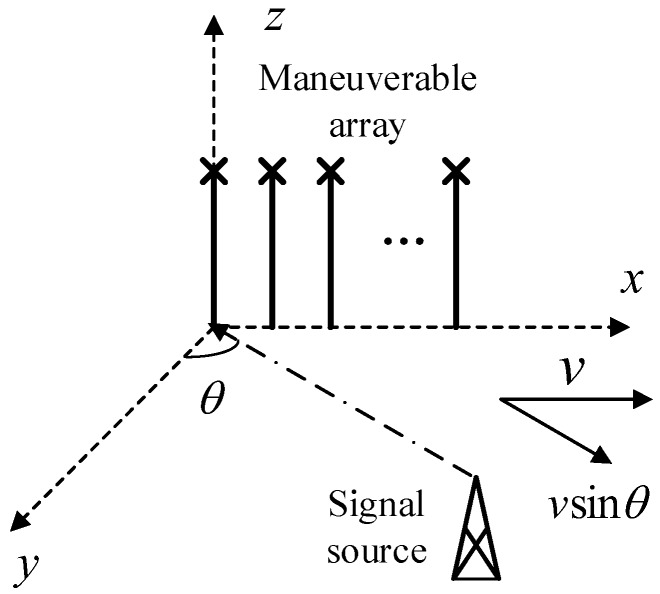
Diagram of moving array.

Assume that a uniform linear array is equipped on a moving platform. The array is composed of M sensors with an inter-element spacing *d* (*d* is equal to half the wavelength), and the platform moves along its baseline at a constant velocity v. The scenario is demonstrated in Figure 1.

We assume that *Q* far-field plane waves impinge on the array from directions $\theta_{q}(q=1,2,\cdots ,Q)$ with frequencies centered at *f*_0_, which is known to the observer. *c* is the velocity of the electromagnetic wave. Because of movement of the array, a Doppler shift $f_{dop}=f_{0}\frac{v\sin\theta }{c}$ is generated and added to the carrier frequency.

Regarding the first sensor as a reference, we can express the observation signal of the *m*-th sensor at time *t* as:

(1) $$\begin{array}{ll} r_{m}(t) & =\sum_{q=1}^{Q}s_{q}(t)\exp\left(j2\pi f_{0}\left(1-\frac{v\sin\theta_{q}}{c}\right)\left(t-\frac{(m-1)d\sin\theta_{q}}{c}\right)\right)+n_{m}(t)\\ & =\sum_{q=1}^{Q}s_{q}(t)\exp\left(j2\pi f_{0}\left(t-\frac{v\sin\theta_{q}}{c}t-\frac{(m-1)d\sin\theta_{q}}{c}+\frac{v(m-1)d\sin^{2}\theta_{q}}{c^{2}}\right)\right)+n_{m}(t) \end{array}$$

where $f_{0}\frac{v\sin\theta_{q}}{c}$ is the Doppler shift, $s_{q}\left(t\right)$ is the signal waveform of the *q*-th source with power $\sigma_{sq}^{2}$, $n_{m}\left(t\right)$ is zero-mean, spatially and temporally white noise with power $\sigma_{n}^{2}$. In addition, the noise is assumed to be uncorrelated with signals, and signals are assumed to be uncorrelated.

Since v≪c, the term $\frac{v(m-1)d\sin^{2}\theta_{q}}{c^{2}}$ in Equation (1) is approximated to zero. Moreover, as $f_{0}$ is known, we can down-convert the observation signal to obtain:

(2) $$x_{m}(t)=\sum_{q=1}^{Q}s_{q}(t)\exp\left(-j2\pi f_{0}\left(\frac{v\sin\theta_{q}}{c}t+\frac{(m-1)d\sin\theta_{q}}{c}\right)\right)+n_{m}(t)$$

where $x_{m}(t)$ represents the observation signal after down-conversion.

From Equation (2), we can find that the time domain samples constitute a geometric sequence like a uniform linear array manifold, so we consider creating a manifold structure in the time domain which is similar to the space domain for obtaining the space-time array.

Supposing that K samples are available, the total samples can be divided into P equal time segments. Every segment has J(J=K/P) samples. To ensure the coherence of signals in a segment, we assume that the duration of a segment is less than the coherence time of the signal. This assumption enables us to ignore the phase fluctuation generated by $s_{q}(t)$ in the same segment.

Then in the *p*-th segment, the observed signal of the *m*-th sensor at the *j*-th sample moment can be expressed as:

(3) $$x_{m,j}(p)=\sum_{q=1}^{Q}s_{q}(p)\exp\left(-j2\pi f_{0}\left(\frac{v\sin\theta_{q}}{c}\left[\left(p-1\right)J+\left(j-1\right)\right]T_{s}+\frac{(m-1)d\sin\theta_{q}}{c}\right)\right)+n_{m,j}(p)$$

where $T_{s}$ represents the sample interval. We can define $l_{q}(p)=\exp\left(-j2\pi f_{0}\frac{v\sin\theta_{q}}{c}\left(p-1\right)JT_{s}\right)$ as the initial phase of the *p*-th segment. Besides, the phase shift of every sample moment during the same segment can make up a vector as follows:

(4) $$b\left(\theta_{q}\right)={\left[1,\;\exp\left(-j2\pi f_{0}\frac{v\sin\theta_{q}}{c}T_{s}\right),\cdots ,\exp\left(-j2\pi f_{0}\frac{v\sin\theta_{q}}{c}\left(J-1\right)T_{s}\right)\right]}^{T}$$

The vector $b\left(\theta_{q}\right)$ is similar to spatial manifold vector $a(\theta_{q})$, so we can recognize $b\left(\theta_{q}\right)$ as the temporal manifold vector and its dimension is J. Then supposing that $z_{m}(p)$ is the observation signal of the *m*-th sensor in the *p*-th segment, we can represent $z_{m}(p)$ as:

(5) $$\begin{array}{ll} z_{m}(p) & =\sum_{q=1}^{Q}s_{q}(p)l_{q}(p)b(\theta_{q})\exp\left(-j2\pi f_{0}\frac{\left(m-1\right)d\sin\theta_{q}}{c}\right)+\xi_{m}(p)\\ & =\sum_{q=1}^{Q}\gamma_{q}(p)b(\theta_{q})\exp\left(-j2\pi f_{0}\frac{\left(m-1\right)d\sin\theta_{q}}{c}\right)+\xi_{m}(p) \end{array}$$

where $\gamma_{q}(p)=s_{q}(p)l_{q}(p)$ is the space-time input of the *q*-th source. $\xi_{m}(p)={\left[n_{m,1}(p),n_{m,2}(p),\cdots ,n_{m,J}(p)\right]}^{T}$ is the space-time noise of the *m*-th sensor. We define $\sigma_{\gamma q}^{2}$ and $\sigma_{\xi }^{2}$ as the power of space-time input and noise, respectively. From the structure of $\gamma_{q}(p)$ and $\xi_{m}(p)$, we can know that $\sigma_{\gamma q}^{2}=\sigma_{sq}^{2}$ and $\sigma_{\xi }^{2}=\sigma_{n}^{2}$. Utilizing the result of Equation (5), we can get an MJ-dimensional space-time array observation vector $z(p)={\left[z_{1}^{T}(p),z_{2}^{T}(p),\cdots ,z_{M}^{T}(p)\right]}^{T}$, which can be written as:

(6) $$\begin{array}{ll} z(p) & =\sum_{q=1}^{Q}\left[a(\theta_{q})⊗b(\theta_{q})\right]\gamma_{q}(p)+\xi (p)\\ & =\sum_{q=1}^{Q}\varphi (\theta_{q})\gamma_{q}(p)+\xi (p) \end{array}$$

where $a(\theta_{q})={\left[1,exp\left(-j2\pi f_{0}\frac{d\sin\theta_{q}}{c}\right),\cdots ,exp\left(-j2\pi f_{0}\frac{\left(M-1\right)d\sin\theta_{q}}{c}\right)\right]}^{T}$ is the spatial manifold vector. $\varphi \left(\theta_{q}\right)=a(\theta_{q})⊗b(\theta_{q})$ is the space-time manifold vector. $\xi (p)={\left[\xi_{1}^{T}(p),\xi_{2}^{T}(p),\cdots ,\xi_{M}^{T}(p)\right]}^{T}$ is the space-time noise vector. ⊗ represents the Kronecker product.

To express Equation (6) in matrix form, we define $A\left(\theta \right)=\left[a(\theta_{1}),a(\theta_{2}),\cdots ,a(\theta_{Q})\right]$, $B\left(\theta \right)=\left[b(\theta_{1}),b(\theta_{2}),\cdots ,b(\theta_{Q})\right]$, $\gamma (p)={\left[\gamma_{1}(p),\gamma_{2}(p),\cdots ,\gamma_{Q}(p)\right]}^{T}$, then the signal model becomes:

(7) $$\begin{array}{ll} z(p) & =\left(A\left(\theta \right)\circ B\left(\theta \right)\right)\gamma (p)+\xi (p)\\ & =\Phi \left(\theta \right)\gamma (p)+\xi (p) \end{array}$$

where ∘ is the Khatri-Rao product. The MJ×Q-dimensional space-time manifold matrix is $\Phi \left(\theta \right)=A\left(\theta \right)\circ B\left(\theta \right)=\left[\varphi (\theta_{1}),\varphi (\theta_{2}),\cdots ,\varphi (\theta_{Q})\right]$.

The modified model transforms temporal samples into virtual sensors, which is equivalent to changing an M sensors uniform linear array (ULA) into an MJ sensors linear array (not always uniform).

## 3. Performance Analysis

This section presents the performance analysis of the space-time array. The comparison with a conventional array is implemented to demonstrate its advantages in the aspects of distinguishable source number, spatial resolution and CRB.

### 3.1. Distinguishable Number of Sources

The number of sources that an array can specify is mainly dependent on its sensor number and the rank of the source sample-correlation matrix. As the equivalent sensor number of a space-time array is enlarged, the distinguishable source number is supposed to increase. We provide a detailed analysis below.

Wax and Ziskind have deduced relevant conclusions in [18]. Their work takes into account the case that signals are correlated and have strong universality. In this paper, we have assumed that signals are uncorrelated. Meanwhile, for simplicity, we suppose that the sample number is sufficient, thus the source sample-correlation matrix is full rank, whose rank is equal to the source number Q. Then using the results from [18], we can easily get that the maximum distinguishable source number of a conventional array is:

(8) $$Q_{\max}=M-1$$

For space-time arrays, the condition is slightly more complicated. The maximum of Q is in the range of the following condition [19]:

(9) $$Q_{\max}\in \left[M+J-2,MJ\right)$$

We note that the distinguishable source number of he space-time array is increased, which coincides with our expectation. However, its maximum is not equal to the virtual sensor number *MJ*, but a range from M+J−2 to MJ. This condition results from the selection of the sample interval *T_s_*(1/*f_f_*). Equation (4) indicates that the equivalent element spacing of b(θ) is $vT_{s}$. When $vT_{s}=d$, the elements of b(θ) will overlap with those of a(θ), which causes that the virtual array φ(θ) has only M+J−1 distinct elements and leads to the least case. On the other hand, when the time domain array is a sparse ULA comprising J elements with interelement spacing Md (i.e., $vT_{s}=Md$), the virtual array is a filled-element (MJ) ULA. This case leads to the result that the maximum number of sources that can be uniquely identified by a space-time array is J times larger than that of a conventional array.

### 3.2. Spatial Resolution

Resolution is one of the important indexes for measuring the array performance. The spatial resolution of an array is closely correlated with the changing rate of the steering vector. To intuitively compare the resolution of different arrays, a scalar η(θ) can be defined to represent the resolving ability [20]:

(10) $$\eta \left(\theta \right)=\left\|\frac{d\mu \left(\theta \right)}{d\theta }\right\|$$

where μ(θ) is the steering vector, ‖·‖ represents the Euclidean norm of a vector.

Here we suppose that $\eta_{O}\left(\theta \right)$ and $\eta_{sp}\left(\theta \right)$ are the resolution of conventional array and space-time array, respectively. To avoid the presence of the cross terms, we set the centers of spatial and temporal steering vector to be the reference point. The final expressions are:

(11) $$\eta_{O}\left(\theta \right)=2\pi f_{0}\frac{\cos\theta }{c}\sqrt{\frac{M}{12}\left[\left(M^{2}-1\right)d^{2}\right]}$$

(12) $$\eta_{sp}\left(\theta \right)=2\pi f_{0}\frac{\cos\theta }{c}\sqrt{\frac{MJ}{12}\left[\left(M^{2}-1\right)d^{2}+\left(J^{2}-1\right){\left(vT_{s}\right)}^{2}\right]}$$

**Proof.**  See Appendix A. □

We can observe that $\eta_{sp}\left(\theta \right)$ contains a term introduced by time domain information in the numerator while $\eta_{O}\left(\theta \right)$ does not contain it. Besides, $\eta_{sp}\left(\theta \right)$ possesses MJ as the coefficient which is J times that of $\eta_{O}\left(\theta \right)$, so the relationship between $\eta_{O}\left(\theta \right)$ and $\eta_{sp}\left(\theta \right)$ is guaranteed to be:

(13) $$\eta_{O}\left(\theta \right)<\eta_{sp}\left(\theta \right)$$

Equation (13) indicates that the space-time array can improve the spatial resolution in the scenario assumed in this paper. Moreover, we can find that $\eta_{sp}\left(\theta \right)$ will increase when $T_{s}$, J or v increases. Hence, longer duration of the time segment and higher velocity are beneficial to the resolution.

### 3.3. CRB

In this section, we derive the DOA estimation CRB of conventional and space-time array model. Then a comparison is provided to provide some in-depth conclusions about the performance.

The derivation of CRB generally relies on random signal (Gaussian) model or nonrandom (conditional) signal model. In this paper, we consider that the sources are nonrandom.

We define $CRB_{O}\left(\theta \right)$ as the CRB of the conventional array model. According to the work of [21], the value of $CRB_{O}\left(\theta \right)$ is computed as:

(14) CRBO(θ)=σn22K{Re[H⊙RsT]}−1

in which ⊙ represents the Hadamard product. $R_{s}=E\left[s\left(k\right)s^{H}\left(k\right)\right]$ is the source covariance matrix. The matrix H is defined as:

$$H=W^{H}\left(\theta \right)\left[I_{M}-A\left(\theta \right){\left(A^{H}\left(\theta \right)A\left(\theta \right)\right)}^{-1}A^{H}\left(\theta \right)\right]W\left(\theta \right)$$

$$W\left(\theta \right)=\left[w\left(\theta_{1}\right),w\left(\theta_{2}\right),\cdots ,w\left(\theta_{Q}\right)\right],\;\left(w\left(\theta_{q}\right)=\frac{\partial a\left(\theta_{q}\right)}{\partial \theta_{q}}\right)$$

where I denotes the identity matrix and the subscript is its dimension.

The CRB of space-time array model can also be obtained in a similar way. From Equations (6) and (7), we know that the dimension of the space-time observation signal z(p) is MJ×P, where MJ is the equivalent sensor number and P is the equivalent sample number. Designating $CRB_{sp}(\theta )$ as the CRB of space-time array model, it can be given by:

(15) CRBsp(θ)=σξ22P{Re[G⊙RγT]}−1

where $R_{\gamma }=E\left[\gamma \left(p\right)\gamma^{H}\left(p\right)\right]$ is the covariance matrix of space-time input. G is similarly defined as H, that is:

$$G=Y^{H}\left(\theta \right)\left[I_{MJ}-\Phi \left(\theta \right){\left(\Phi^{H}\left(\theta \right)\Phi \left(\theta \right)\right)}^{-1}\Phi^{H}\left(\theta \right)\right]Y\left(\theta \right)$$

$$Y\left(\theta \right)=\left[y\left(\theta_{1}\right),y\left(\theta_{2}\right),\cdots ,y\left(\theta_{Q}\right)\right],\;\left(y\left(\theta_{q}\right)=\frac{\partial \varphi \left(\theta_{q}\right)}{\partial \theta_{q}}\right)$$

To be convenient to make contrast between the CRB of two models, $CRB_{O}\left(\theta \right)$ and $CRB_{sp}\left(\theta \right)$ can be further deduced to become:

(16) $$\begin{array}{l} CRB_{O}\left(\theta \right)\approx \frac{6}{MK}diag\left\{\begin{array}{cccc} \alpha_{1} & \alpha_{2} & \cdots & \alpha_{Q} \end{array}\right\}\\ \alpha_{q}=\frac{1}{SNR_{q}^{}{\left(2\pi f_{0}\frac{\cos\theta_{q}}{c}\right)}^{2}\left[\left(M^{2}-1\right)d^{2}\right]}\;\;\;q=1,\cdots ,Q \end{array}$$

(17) $$\begin{array}{l} CRB_{sp}\left(\theta \right)\approx \frac{6}{MK}diag\left\{\begin{array}{cccc} \beta_{1} & \beta_{2} & \cdots & \beta_{Q} \end{array}\right\}\\ \beta_{q}=\frac{1}{SNR_{q}{\left(2\pi f_{0}\frac{\cos\theta_{q}}{c}\right)}^{2}\left[\left(M^{2}-1\right)d^{2}+\left(J^{2}-1\right){\left(vT_{s}\right)}^{2}\right]}\;\;\;q=1,\cdots ,Q \end{array}$$

In Equations (16) and (17), $SNR_{q}$ is the signal-to-noise ratio (SNR) of the *q*-th signal and its value is $\sigma_{sq}^{2}/\sigma_{n}^{2}$ (equal to $\sigma_{\gamma q}^{2}/\sigma_{\xi }^{2}$).

**Proof.**  See Appendix B. □

It is worth noting that $CRB_{sp}\left(\theta \right)$ contains a time domain information term in the denominator. To concisely make comparison between $CRB_{O}\left(\theta \right)$ and $CRB_{sp}\left(\theta \right)$, we take the single signal case as the example. Then the relationship between the CRB of the two array models can be expressed as:

(18) $$CRB_{sp}^{-1}\left(\theta \right)=CRB_{O}^{-1}\left(\theta \right)+\frac{MKSNR{\left(2\pi f_{0}\frac{\cos\theta }{c}\right)}^{2}\left(J^{2}-1\right){\left(vT_{s}\right)}^{2}}{6}$$

Obviously, when J>1 and v>0, the temporal array can bring array gain and yield $CRB_{sp}^{}\left(\theta \right)<CRB_{O}^{}\left(\theta \right)$, which demonstrates that the space-time array model can reduce the CRB. Meanwhile, the increase of $T_{s}$, J or v can enlarge the temporal array gain and lead to lower CRB.

**Remark** **1.** As concluded above, $T_{s}$ and J are important factors impacting the performance. However, Equation (4) indicates that the coherent processing interval is $JT_{s}$. This duration is subject to the signal coherent time. Thus parameter $T_{s}$ and J cannot increase infinitely and should be carefully selected based on signal temporal coherence.

**Remark** **3.** The condition $vT_{s}=Md$ can lead to optimal distinguishable source number MJ. Nonetheless, the increase of $T_{s}$ may decrease J when the signal coherent time is unchanged, which makes the improvement of the distinguishable source number inconspicuous. Hence it is suggested to enlarge v for multi-signal cases.

**Remark** **3.** Expressions of resolution and CRB have similar forms. $\eta_{sp}\left(\theta \right)$ has a time domain information term in the numerator while $CRB_{sp}\left(\theta \right)$ has this term in the denominator. Therefore, from the perspective of expressions, enhancement results from the simultaneous employment of spatial and temporal information.

**Remark** **4.** $\eta_{sp}\left(\theta \right)$ is mainly impacted by the temporal manifold vector dimension J since it possesses J as the coefficient rather than $JT_{s}$. Therefore, we can properly enlarge J and decrease $T_{s}$ when we need higher spatial resolution, and this operation will not degrade the DOA estimation accuracy (CRB preserved).

## 4. Simulation

In this section, some computer simulations are presented to verify our deduction about space-time arrays and demonstrate its performance. In the figures of this section, the curves with postfix ‘conventional’ and ‘ST’ denote the performance curves of a conventional array and a space-time array, respectively. We consider a uniformly linear array with nine sensors. The array is equipped on an airplane and moves along its baseline at a constant velocity of 200 m/s.

**Figure 2:**
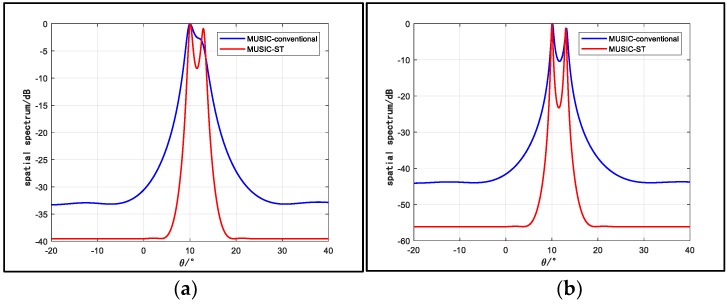
Spatial spectrum for different SNRs: (**a**) SNR = 2dB; (**b**) SNR = 10dB.

First, we resort to the MUSIC algorithm to check the resolving ability of the space-time array (the specific operations of the algorithm can be seen in Appendix C). Assume that two narrow-band signals are impinging on the array from angles of 10° and 13°, respectively. The carrier frequency is 300 MHz. The dimension of the temporal manifold vector is 20 (J = 20). The sample number is 1200 and the sample interval ($T_{s}$) after down-conversion is set to satisfy $vT_{s}=d$. Figure 2 displays the spatial spectrum of two arrays for different SNRs. In Figure 2a, only the space-time array can distinguish the two sources. In Figure 2b, when the SNR is high, the two arrays can both distinguish them.

**Figure 3:**
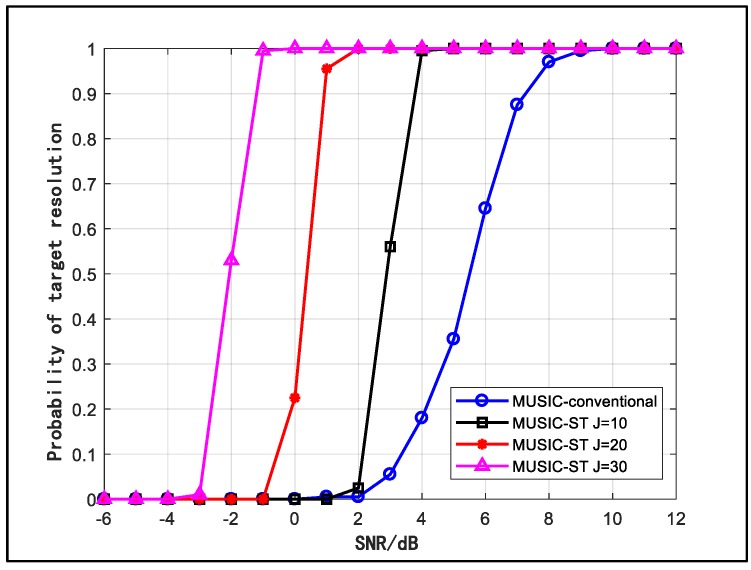
Probability of target resolution versus SNR.

To further demonstrate the spatial resolution enhancement, we plot the probability of the target resolution curves for different values of J in Figure 3, where the input SNR changes from −6 dB to 12 dB and other conditions are maintained. From the figure, we can see that the space-time array has better source resolution capability than the conventional array especially in the low SNR region, and the result appears to achieve higher probability of target resolution with larger J. Hence, the array spatial resolution can be enhanced by involving more virtual antennas. The results in Figure 2 and Figure 3 indicate that the capability of resolving adjacent sources of a space-time array is much better than that of a conventional array.

**Figure 4:**
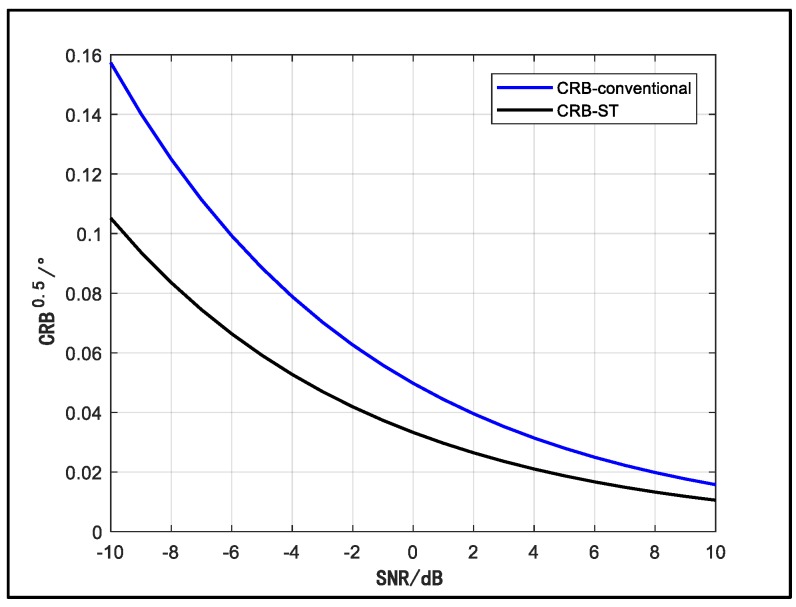
CRB of signal direction estimation versus SNR.

**Figure 5:**
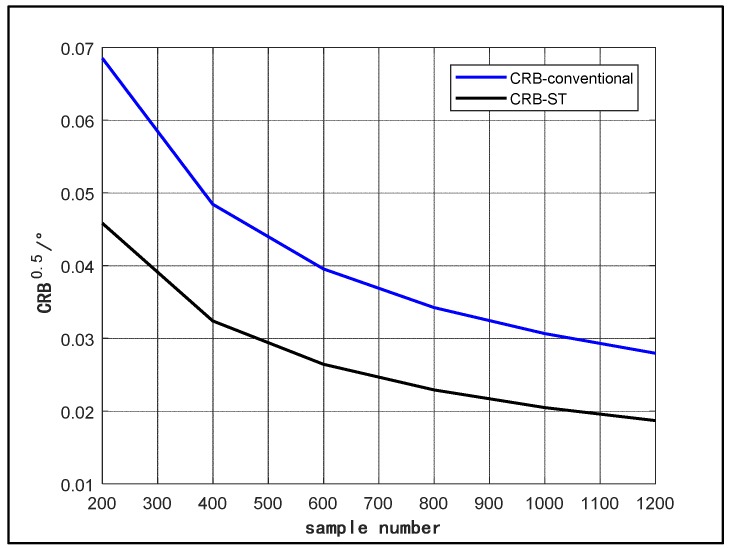
CRB of signal direction estimation versus sample number.

Next, we verify the deduction about CRB. Figure 4 and Figure 5 show the contrast between the CRB of the conventional and space-time array model over SNR and sample number, respectively. In Figure 4, we assume that a signal is impinging on the array from 15°. The signal carrier frequency, sample number, sample interval and platform moving velocity stay the same as the scenario in Figure 2 and Figure 3. The dimension of the temporal manifold vector is 10 (J = 10). The SNR changes from from −10 dB to 10 dB. In Figure 5, we fix SNR at a constant value of 5 dB. The sample number changes from 200 to 1200. Other conditions are the same as Figure 4. From the two figures, it can be observed that the CRB of the space-time array is obviously lower than that of the conventional array, which indicates that the space-time array can improve the estimation accuracy.

**Figure 6:**
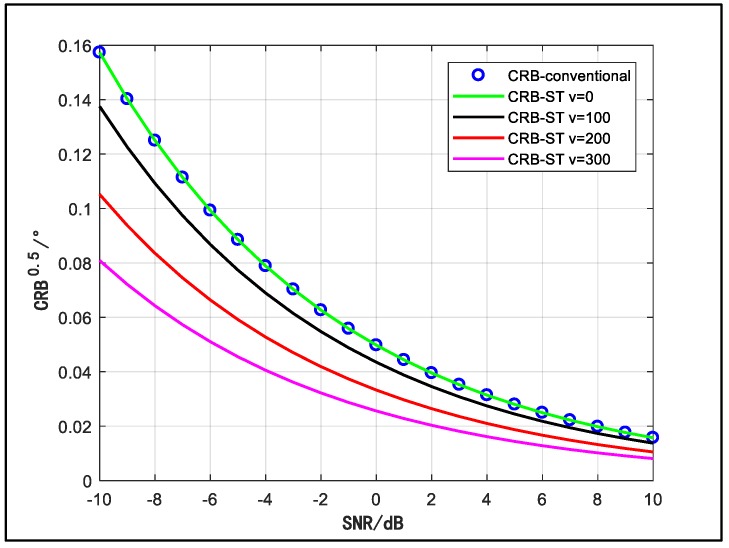
CRB versus SNR for different values of the velocity.

Figure 6 shows the CRB performance of the space-time array for different velocities. We set the same scenario as Figure 4, and the signal carrier frequency, sample number and sample interval are also unchanged. The dimension of b(θ) is 10 (J = 10). We can observe that the CRB performance improved with the velocity gets larger. When the velocity reaches 300 m/s, the CRB can reach below 0.01° for high SNR. Besides, for low SNR, the improvement is more obvious.

**Figure 7:**
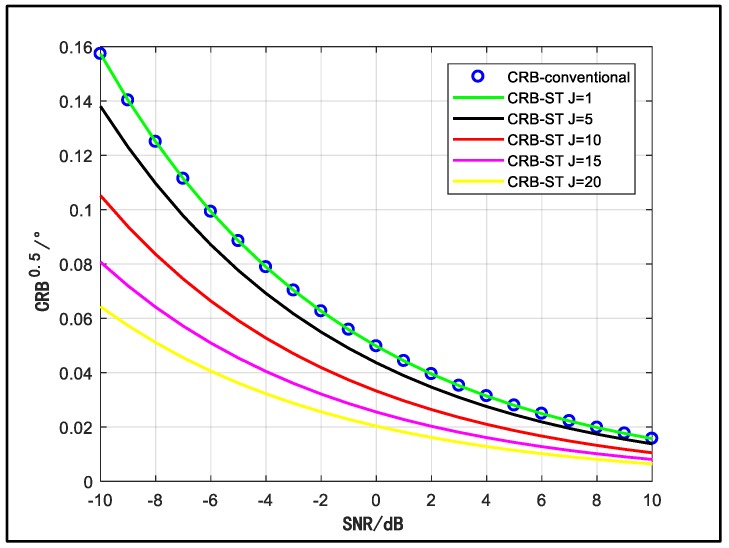
CRB versus SNR for different values of the temporal dimension.

Like Figure 6, we depict five curves to represent different temporal manifold vector dimensions in Figure 7. The velocity is 200 m/s and other conditions stay the same. It can be seen that the performance is better with more equivalent temporal array elements, but the improvement tendency becomes slower with enlarged J. Besides, we know that the complexity of the space-time array will extend when J increases, so it is important to make a compromise between complexity and performance for the selection of J.

In Figure 6 and Figure 7, specially, when v = 0 or J = 1, the performance curve is coincident with the curve of the conventional array. Under the condition that v = 0, i.e., the array is static, there is no Doppler shift in the signal time domain. The temporal manifold vector $b\left(\theta \right)={\left[1,1,\cdots ,1\right]}^{T}$, which does not contain DOA information. Therefore, although the dimension of the space-time manifold vector is enlarged, the available information is not increased so that the performance is not improved. On the other hand, for the case that the signal has relatively wide bandwidth, whose coherent time is short, the number of the samples in a time segment is limited. In the worst case (i.e., J = 1), the dimension of the array steering vector is not extended, which means that the DOA information in time domain is not employed, so the performance curve also converges to the curve of the conventional array.

## 5. Conclusions

This paper constructs a joint space-time array model based on a moving platform. The proposed space-time array combines spatial information with temporal information and obtains better performance than a conventional array, which makes it applicable in those conditions assumed in this paper. The theoretical analysis demonstrates that the space-time array improves the distinguishable source number, spatial resolution and estimation accuracy. Numerical simulations validate our deduction and illustrate the reliability of the space-time array performance. Nevertheless, our method requires that the duration of the time segment be less than the coherence time of the signal. When the signal bandwidth is relatively wide, the performance of proposed space-time array will degrade severely. Thus, our future work will focus on developing methods for more general environment.

## Appendix A

Derivation of $\eta_{O}\left(\theta \right)$ and $\eta_{sp}\left(\theta \right)$ According to the statement in Section 3.2, the spatial and temporal steering vector can be written as:

$$\begin{array}{ll} a_{\eta }(\theta ) & =\exp\left(j2\pi f_{0}\frac{d\sin\theta }{c}\left(-\frac{M-1}{2}\right)\right)a(\theta )\\ & ={\left[\exp\left(j2\pi f_{0}\frac{d\sin\theta }{c}\left(-\frac{M-1}{2}\right)\right),\cdots ,\exp\left(j2\pi f_{0}\frac{d\sin\theta }{c}\left(\frac{M-1}{2}\right)\right)\right]}^{T} \end{array}$$

$$\begin{array}{ll} b_{\eta }\left(\theta \right) & =\exp\left(j2\pi f_{0}\frac{v\sin\theta }{c}\left(-\frac{J-1}{2}\right)T_{s}\right)b\left(\theta \right)\\ & ={\left[\exp\left(j2\pi f_{0}\frac{v\sin\theta }{c}\left(-\frac{J-1}{2}\right)T_{s}\right),\cdots ,\exp\left(j2\pi f_{0}\frac{v\sin\theta }{c}\left(\frac{J-1}{2}\right)T_{s}\right)\right]}^{T} \end{array}$$

respectively.

The derivative of $a_{\eta }\left(\theta \right)$ is:

$$\begin{array}{c} w_{\eta }\left(\theta \right)=\frac{da_{\eta }\left(\theta \right)}{d\theta }=[j2\pi f_{0}\frac{d\cos\theta }{c}\left(-\frac{M-1}{2}\right)e^{j2\pi f_{0}\frac{d\sin\theta }{c}\left(-\frac{M-1}{2}\right)},\cdots ,\\ {j2\pi f_{0}\frac{d\cos\theta }{c}\left(\frac{M-1}{2}\right)e^{j2\pi f_{0}\frac{d\sin\theta }{c}\left(\frac{M-1}{2}\right)}]}^{T} \end{array}$$

Then calculating the Euclidean norm of $w_{\eta }\left(\theta \right)$, we can get:

(A1) $$\eta_{O}\left(\theta \right)={\left\|w_{\eta }\left(\theta \right)\right\|}_{2}=2\pi f_{0}\frac{\cos\theta }{c}\sqrt{\frac{M}{12}\left[\left(M^{2}-1\right)d^{2}\right]}$$

The acquirement of $\eta_{O}\left(\theta \right)$ has been proved. Next we deduce $\eta_{sp}\left(\theta \right)$.

First, we should calculate the space-time manifold vector:

$$\begin{array}{ll} \varphi_{\eta }(\theta )=a_{\eta }(\theta )⊗b_{\eta }(\theta )= & [\exp\left(j2\pi f_{0}\left(-\frac{M-1}{2}\frac{d\sin\theta }{c}-\frac{J-1}{2}\frac{v\sin\theta }{c}T_{s}\right)\right),\cdots ,\\ & \exp\left(j2\pi f_{0}\left(-\frac{M-1}{2}\frac{d\sin\theta }{c}+\frac{J-1}{2}\frac{v\sin\theta }{c}T_{s}\right)\right),\cdots ,\\ & \exp\left(j2\pi f_{0}\left(\frac{M-1}{2}\frac{d\sin\theta }{c}-\frac{J-1}{2}\frac{v\sin\theta }{c}T_{s}\right)\right),\cdots ,\\ & {\exp\left(j2\pi f_{0}\left(\frac{M-1}{2}\frac{d\sin\theta }{c}+\frac{J-1}{2}\frac{v\sin\theta }{c}T_{s}\right)\right)]}^{T} \end{array}$$

Then according to the same step as $\eta_{O}\left(\theta \right)$, $\eta_{sp}\left(\theta \right)$ can be obtained as follow:

(A2) $$\eta_{sp}\left(\theta \right)={\left\|\frac{d\varphi_{\eta }\left(\theta \right)}{d\theta }\right\|}_{2}=2\pi f_{0}\frac{\cos\theta }{c}\sqrt{\frac{MJ}{12}\left[\left(M^{2}-1\right)d^{2}+\left(J^{2}-1\right){\left(vT_{s}\right)}^{2}\right]}$$

## Appendix B

Derivation of $CRB_{O}\left(\theta \right)$ and $CRB_{sp}^{}\left(\theta \right)$ In this Appendix we use the steering vectors given in Section 2.1. We can compute $w\left(\theta_{q}\right)$ utilizing $a\left(\theta_{q}\right)$ and obtain:

$$w\left(\theta_{q}\right)=\frac{da\left(\theta_{q}\right)}{d\theta_{q}}={\left[0,-j2\pi f_{0}\frac{d\cos\theta_{q}}{c}e^{-j2\pi f_{0}\frac{d\sin\theta_{q}}{c}},\cdots ,-j2\pi f_{0}\frac{\left(M-1\right)d\cos\theta_{q}}{c}e^{-j2\pi f_{0}\frac{\left(M-1\right)d\sin\theta_{q}}{c}}\right]}^{T}$$

The matrix H can be written as:

$$\begin{array}{ll} H & =W^{H}\left(\theta \right)\left[I_{M}-A\left(\theta \right){\left(A^{H}\left(\theta \right)A\left(\theta \right)\right)}^{-1}A^{H}\left(\theta \right)\right]W\left(\theta \right)\\ & =W^{H}\left(\theta \right)W\left(\theta \right)-W^{H}\left(\theta \right)A\left(\theta \right){\left(A^{H}\left(\theta \right)A\left(\theta \right)\right)}^{-1}A^{H}\left(\theta \right)W\left(\theta \right) \end{array}$$

Some approximations are necessary to be made in the derivation process.

Before calculating H, we need to get the following results firstly [22]:

$$A^{H}\left(\theta \right)A\left(\theta \right)\approx diag{\left\{M,M,\cdots ,M\right\}}_{Q\times Q}$$

$$W^{H}\left(\theta \right)W\left(\theta \right)\approx \frac{M\left(M-1\right)\left(2M-1\right)}{6}diag\left\{{\left(2\pi f_{0}\frac{d}{c}\cos\theta_{1}\right)}^{2},{\left(2\pi f_{0}\frac{d}{c}\cos\theta_{2}\right)}^{2},\cdots ,{\left(2\pi f_{0}\frac{d}{c}\cos\theta_{Q}\right)}^{2}\right\}$$

$$W^{H}\left(\theta \right)A\left(\theta \right)\approx j\frac{M\left(M-1\right)}{2}diag\left\{2\pi f_{0}\frac{d}{c}\cos\theta_{1},2\pi f_{0}\frac{d}{c}\cos\theta_{2},\cdots ,2\pi f_{0}\frac{d}{c}\cos\theta_{Q}\right\}$$

Then we can obtain that H is:

(A3) $$H\approx \frac{M\left(M^{2}-1\right)}{12}diag\left\{{\left(2\pi f_{0}\frac{d}{c}\cos\theta_{1}\right)}^{2},{\left(2\pi f_{0}\frac{d}{c}\cos\theta_{2}\right)}^{2},\cdots ,{\left(2\pi f_{0}\frac{d}{c}\cos\theta_{Q}\right)}^{2}\right\}$$

Next we consider the other part of Equation (14). In this part:

(A4) $$R_{s}=\frac{1}{K}\sum_{k=1}^{K}s\left(k\right)s^{H}\left(k\right)=diag\left\{\sigma_{s1}^{2},\sigma_{s2}^{2},\cdots ,\sigma_{sQ}^{2}\right\}$$

Substituting Equations (A3) and (A4) into Equation (14), we can obtain Equation (16).

The derivation of $CRB_{sp}^{}\left(\theta \right)$ follows the same procedure as $CRB_{O}\left(\theta \right)$. First, we should respectively obtain $\varphi (\theta_{q})$ and $y(\theta_{q})$ as follows:

$$\begin{array}{l} \varphi (\theta_{q})=a(\theta_{q})⊗b(\theta_{q})=[1,e^{-j2\pi f_{0}\frac{v\sin\theta_{q}}{c}T_{s}},\cdots ,e^{-j2\pi f_{0}\frac{v\sin\theta_{q}}{c}\left(J-1\right)T_{s}},\cdots ,\\ e^{-j2\pi f_{0}\frac{d\sin\theta_{q}}{c}},e^{-j2\pi f_{0}\left(\frac{d\sin\theta_{q}}{c}+\frac{v\sin\theta_{q}}{c}T_{s}\right)},\cdots ,e^{-j2\pi f_{0}\left(\frac{d\sin\theta_{q}}{c}+\frac{v\sin\theta_{q}}{c}\left(J-1\right)T_{s}\right)},\cdots ,\\ {e^{-j2\pi f_{0}\frac{\left(M-1\right)d\sin\theta_{q}}{c}},e^{-j2\pi f_{0}\left(\frac{\left(M-1\right)d\sin\theta_{q}}{c}+\frac{v\sin\theta_{q}}{c}T_{s}\right)},\cdots ,e^{-j2\pi f_{0}\left(\frac{\left(M-1\right)d\sin\theta_{q}}{c}+\frac{v\sin\theta_{q}}{c}\left(J-1\right)T_{s}\right)}]}^{T} \end{array}$$

$$\begin{array}{l} y(\theta_{q})=\frac{d\varphi (\theta_{q})}{d\theta_{q}}=[0,-j2\pi f_{0}\frac{v\cos\theta_{q}}{c}T_{s}e^{-j2\pi f_{0}\frac{v\sin\theta_{q}}{c}T_{s}},\cdots ,-j2\pi f_{0}\frac{v\cos\theta_{q}}{c}\left(J-1\right)T_{s}e^{-j2\pi f_{0}\frac{v\sin\theta_{q}}{c}\left(J-1\right)T_{s}},\cdots ,\\ -j2\pi f_{0}\frac{d\cos\theta_{q}}{c}e^{-j2\pi f_{0}\frac{d\sin\theta_{q}}{c}},-j2\pi f_{0}\left(\frac{d\cos\theta_{q}}{c}+\frac{v\cos\theta_{q}}{c}T_{s}\right)e^{-j2\pi f_{0}\left(\frac{d\sin\theta_{q}}{c}+\frac{v\sin\theta_{q}}{c}T_{s}\right)},\cdots ,\\ -j2\pi f_{0}\left(\frac{d\cos\theta_{q}}{c}+\frac{v\cos\theta_{q}}{c}\left(J-1\right)T_{s}\right)e^{-j2\pi f_{0}\left(\frac{d\sin\theta_{q}}{c}+\frac{v\sin\theta_{q}}{c}\left(J-1\right)T_{s}\right)},\cdots ,-j2\pi f_{0}\frac{\left(M-1\right)d\cos\theta_{q}}{c}e^{-j2\pi f_{0}\frac{\left(M-1\right)d\sin\theta_{q}}{c}},\\ -j2\pi f_{0}\left(\frac{\left(M-1\right)d\cos\theta_{q}}{c}+\frac{v\cos\theta_{q}}{c}T_{s}\right)e^{-j2\pi f_{0}\left(\frac{\left(M-1\right)d\sin\theta_{q}}{c}+\frac{v\sin\theta_{q}}{c}T_{s}\right)},\cdots ,\\ {-j2\pi f_{0}\left(\frac{\left(M-1\right)d\cos\theta_{q}}{c}+\frac{v\cos\theta_{q}}{c}\left(J-1\right)T_{s}\right)e^{-j2\pi f_{0}\left(\frac{\left(M-1\right)d\sin\theta_{q}}{c}+\frac{v\sin\theta_{q}}{c}\left(J-1\right)T_{s}\right)}]}^{T} \end{array}$$

Then the matrix G can be calculated analogously to matrix H with the knowledge of $\varphi (\theta_{q})$ and $y(\theta_{q})$:

(A5) $$\begin{array}{ll} G\approx & diag\{\frac{MJ\left(J^{2}-1\right)}{12}{\left(2\pi f_{0}\frac{v\cos\theta_{1}}{c}T_{s}\right)}^{2}+\frac{MJ\left(M^{2}-1\right)}{12}{\left(2\pi f_{0}\frac{d\cos\theta_{1}}{c}\right)}^{2},\\ & \frac{MJ\left(J^{2}-1\right)}{12}{\left(2\pi f_{0}\frac{v\cos\theta_{2}}{c}T_{s}\right)}^{2}+\frac{MJ\left(M^{2}-1\right)}{12}{\left(2\pi f_{0}\frac{d\cos\theta_{2}}{c}\right)}^{2},\cdots ,\\ & \frac{MJ\left(J^{2}-1\right)}{12}{\left(2\pi f_{0}\frac{v\cos\theta_{Q}}{c}T_{s}\right)}^{2}+\frac{MJ\left(M^{2}-1\right)}{12}{\left(2\pi f_{0}\frac{d\cos\theta_{Q}}{c}\right)}^{2}\} \end{array}$$

The value of the other factor in Equation (15) is:

(A6) $$R_{\gamma }=\frac{1}{P}\sum_{p=1}^{P}\gamma \left(p\right)\gamma^{H}\left(p\right)=diag\left\{\sigma_{\gamma 1}^{2},\sigma_{\gamma 2}^{2},\cdots ,\sigma_{\gamma Q}^{2}\right\}$$

Substituting Equations (A5) and (A6) into Equation (15), we can obtain Equation (17).

## Appendix C

MUSIC algorithm for resolution comparison

To intuitively demonstrate the spatial resolution comparison between conventional and space-time array, we employ MUSIC algorithm. The detailed steps are shown below.

First, we make an eigenvalue decomposition of the covariance matrixes and obtain:

(A7) $$R_{x}=U_{s}\Sigma_{s}U_{s}^{H}+U_{n}\Sigma_{n}U_{n}^{H}$$

(A8) $$R_{z}=U_{\gamma }\Sigma_{\gamma }U_{\gamma }^{H}+U_{\xi }\Sigma_{\xi }U_{\xi }^{H}$$

where $U_{s}$ contains the signal subspace eigenvectors of $R_{x}$ and diagonal matrix $\Sigma_{s}$ contains the corresponding eigenvalues. Similarly $U_{\gamma }$ contains the signal subspace eigenvectors of $R_{z}$ and diagonal matrix $\Sigma_{\gamma }$ contains corresponding eigenvalues. $U_{n}$ and $U_{\xi }$ contain the noise eigenvectors, meanwhile $\Sigma_{n}$ and $\Sigma_{\xi }$ contain the eigenvalues. The MUSIC spatial spectrum is computed by:

(A9) $${\hat{f}}_{MUSIC}\left(\theta \right)=\frac{1}{a^{H}\left(\theta \right)U_{n}U_{n}^{H}a\left(\theta \right)}$$

(A10) $${\hat{f}}_{MUSIC-ST}\left(\theta \right)=\frac{1}{\varphi^{H}\left(\theta \right)U_{\xi }U_{\xi }^{H}\varphi \left(\theta \right)}$$

where ${\hat{f}}_{MUSIC}\left(\theta \right)$ and ${\hat{f}}_{MUSIC-ST}\left(\theta \right)$ represent the values of the MUSIC spectrum based on conventional model and space-time model, respectively.

We can utilize Equations (A9) and (A10) to plot the spatial spectrum and observe the circumstance of distinguishing adjacent signals.
